# Review: Modulating the unfolded protein response to prevent neurodegeneration and enhance memory

**DOI:** 10.1111/nan.12211

**Published:** 2015-05-13

**Authors:** Mark Halliday, Giovanna R. Mallucci

**Affiliations:** ^1^ MRC Toxicology UnitHodgkin BuildingUniversity of LeicesterLeicesterUK; ^2^ Department of Clinical NeurosciencesSchool of Clinical MedicineUniversity of CambridgeAddenbrookes HospitalCambridgeUK

**Keywords:** memory, neurodegeneration, neurodegenerative diseases, therapeutics, unfolded protein response

## Abstract

Recent evidence has placed the unfolded protein response (UPR) at the centre of pathological processes leading to neurodegenerative disease. The translational repression caused by UPR activation starves neurons of the essential proteins they need to function and survive. Restoration of protein synthesis, via genetic or pharmacological means, is neuroprotective in animal models, prolonging survival. This is of great interest due to the observation of UPR activation in the *post mortem* brains of patients with Alzheimer's, Parkinson's, tauopathies and prion diseases. Protein synthesis is also an essential step in the formation of new memories. Restoring translation in disease or increasing protein synthesis from basal levels has been shown to improve memory in numerous models. As neurodegenerative diseases often present with memory impairments, targeting the UPR to both provide neuroprotection and enhance memory provides an extremely exciting novel therapeutic target.

## Introduction: the protein misfolding disorders

The development of disease‐modifying therapies for neurodegenerative diseases remains one of the biggest challenges facing society worldwide. As life expectancy increases, a concurrent rise in neurodegenerative diseases is occurring globally as the population ages. These disorders, which include Alzheimer's disease (AD), Parkinson's disease (PD), frontotemporal dementia (FTD) and tauopathies, as well as amyotrophic lateral sclerosis (ALS) and prion diseases, have distinct clinical, pathological and biochemical signatures, and all involve the accumulation of disease‐specific misfolded proteins in the brain. They are now collectively termed protein misfolding disorders [1]. At the molecular level, abnormally folded proteins include oligomers, aggregates or large‐protein inclusions. A great deal of research effort has been directed to unraveling how each individual ‘toxic’ protein exerts its deleterious effects in specific diseases, but to date, mechanistic insights into how these cause neuronal loss has been limited and consequently therapeutic advances have been elusive.

AD is the most common cause of dementia and is characterized by episodic memory loss, progressive cognitive impairment and behavioural changes. Pathologically, amyloid plaques and neurofibrillary tangles are seen in *post mortem* brains. Amyloid plaques are mainly composed of amyloid‐β (Aβ) peptides, typically 1–40 and 1–42 amino acids in size, that are produced from the cleavage of the amyloid precursor protein (APP) by secretases [2]. Hyperphosphorylated tau protein misfolds and aggregates to form neurofibrillary tangles [3]. PD is characterized by extra‐pyramidal motor symptoms and signs, caused by the loss of dopaminergic neurons in the substantia nigra with variable dementia [4]. Aggregated α‐synuclein is the major component of Lewy bodies, abnormal aggregates of protein that are characteristic of PD [5]. ALS is a progressive paralytic disease, involving the selective degeneration of motor neurons in the central and peripheral nervous systems that eventually leads to breathing failure. Several misfolded proteins have been linked to ALS, including superoxide dismutase 1 (SOD1), TAR DNA‐binding protein 43 KDa (TDP‐43) and fused in sarcoma [6]. The prototypic, but rarest, of these disorders are the prion diseases, which include Creutzfeldt–Jakob disease (CJD). These are characterized clinically by rapidly progressive dementia and movement disorders, and pathologically by spongiform degeneration of the brain and the accumulation of protease‐resistant prion protein (PrP) [7].

It is increasingly clear that this group of diseases have common features as well as specific characteristics. Prion diseases are transmissible, and the mechanism of infectivity and spread involves conversion of native PrP (PrP^C^) by the misfolded form, termed PrP^Sc^, via an auto‐catalytic post‐translational change in conformation. As neurons become depleted of PrP^C^, newly synthesized PrP^C^ is produced, providing additional substrate for the conversion. PrP^Sc^ can spread between neurons, gradually increasing the area of the brain affected, or between organisms if infected material is ingested or transferred by iatrogenic exposure. It is now apparent that Aβ, tau and α‐synuclein are capable of the spreading templated conformational change first described for prions. It has been shown that this can cause spread between cells in vitro [8, 9, 10] and also in mouse models, where spread of these proteins can cause regional pathology and disease progression [11, 12, 13]. Recently, the propagation of wild‐type α‐synuclein causing sporadic phenotypes has been reported, as has transmission between animals [13, 14]. However, despite the universality of the prion‐like spreading phenomenon, not all of these models show associated neurodegeneration, especially when transferred between animals [15]. Nevertheless, this raises the concept that protein misfolding disorders have two aspects, cell autonomous processes that cause cellular dysfunction and neurodegeneration, and non‐cell autonomous processes through which the misfolded proteins, and pathology, spreads.

Another common theme in these disorders is endoplasmic reticulum (ER) stress [16]. The ER controls the different cellular processes by which many proteins are synthesized, folded, modified and transported to their intended destinations. Disturbance in the function of the ER leads to ER stress and is usually caused by accumulation of unfolded proteins and by changes in calcium homeostasis within the ER [17]. In neurodegenerative diseases, many of the cellular processes controlled by the ER can be disrupted by the disease pathology, causing stress that contributes and exacerbates neuronal cell death [16]. The cell's main weapon against ER stress is induction of the unfolded protein response (UPR), which has recently emerged as an extremely interesting and attractive therapeutic target [18], due to its role in memory and neurodegeneration.

## The UPR

**Figure 1:**
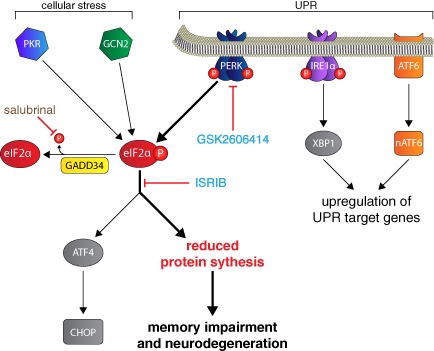
UPR and ISR signalling through eIF2α‐P. Unfolded proteins induce the induction of the UPR, which signals through PERK, IRE1 and ATF6. PERK phosphorylates eIF2α, leading to the rapid attenuation of protein synthesis. Chronic reduction in protein synthesis can lead to memory impairment and neurodegeneration. eIF2α‐P also leads to the selective translation of some proteins such as ATF4 and the pro‐apoptotic CHOP. Other kinases activated by cellular stress, such as PKR and GCN2, can also phosphorylate eIF2α. GADD34 dephosphorylates eIF2α‐P, restoring translation to normal levels. The PERK inhibitor GSK2606414 and the compound ISRIB prevent neurodegeneration and improve memory, respectively. Salubrinal inhibits eIF2α‐P dephosphorylation, exacerbating neurodegeneration in some, but not all models. ATF4, activating transcription factor 4; ATF6, activating transcription factor 6; eIF2α‐P, phosphorylated eIF2α; GNC2, general control non‐derepressible‐2; IRE1, inositol‐requiring enzyme 1; ISR, integrated stress response; PERK, PKR‐like endoplasmic reticulum kinase; PKR, protein kinase RNA; UPR, unfolded protein response; XBP1, x‐box binding protein 1.

The UPR is a protective cellular response induced during periods of ER stress that aims to reduce unfolded protein load and restore protein‐folding homeostasis, ‘proteostasis’. Secreted and transmembrane proteins enter the ER as unfolded proteins to be properly assembled or to be targeted for degradation [19]. During ER stress, proteins inside the ER lumen waiting to be folded and exported build up, causing a backlog that is stressful to the cell. This is detected by binding immunoglobulin protein (BiP), which binds to exposed hydrophobic domains of unfolded proteins [20]. The UPR has three arms (Figure 1), which initiates signalling cascades through protein kinase RNA (PKR)‐like ER kinase (PERK), inositol‐requiring enzyme 1 (IRE1) and activating transcription factor 6 (ATF6) [18]. BiP holds these proteins in an inactive state until it binds unfolded proteins and dissociates, allowing their activation. The cells' first response to ER stress is to attenuate further protein synthesis until the unfolded protein backlog is removed. This is controlled by PERK, which when freed from BiP, dimerizes and autophosphorylates [21]. Phosphorylated PERK (PERK‐P) will then phosphorylate eIF2α, rapidly and potently attenuating translation [22]. eIF2α is a vital component of ternary complex, which loads mRNA onto the ribosome during the initiation of translation [23]. The energy for this reaction is supplied in the form of guanosine‐5′‐triphosphate (GTP) by eIF2B. Phosphorylated eIF2α (eIF2α‐P) binds so tightly to eIF2B that it can no longer recycle GTP, and due to the overabundance of eIF2α compared with eIF2B, even a small increase in eIF2α‐P will greatly inhibit ternary complex formation and hence translation [23].

Paradoxically, the translation of some proteins is increased after eIF2α phosphorylation, due to the presence of multiple open reading frames in their 5′ untranslated regions [24]. One such protein is activating transcription factor 4 (ATF4) [25]. ATF4 controls the expression of various genes involved in apoptosis, autophagy, amino acid metabolism and antioxidant responses. Excessive UPR activation causes ATF4 to induce the transcription of the pro‐apoptotic C/EBP homologous protein (CHOP). There are also three other kinases that can phosphorylate eIF2α, each of which is activated by a different cellular stress: the double‐stranded RNA‐activated protein kinase (PKR) responds to viral infection, general control non‐derepressible‐2 (GCN2) is activated during amino acid starvation and the heme‐regulated inhibitor kinase responds to heme deficiency. Cytoprotective signalling through these eIF2α kinases is termed the integrated stress response [26]. After the ER stress has been resolved and any unfolded proteins have been removed, the translational repression is reversed by dephosphorylation of eIF2α by the phosphatase GADD34 [27].

The IRE1 and ATF6 arms of the UPR aim to increase the protein folding ability of the cell via the induction of chaperones and facilitate the removal of terminally misfolded proteins. Activation of IRE1 by dimerization and autophosphorylation enables its endoribonuclease activity and catalyses the splicing of x‐box binding protein 1 (XBP1) mRNA [28]. This splicing causes a frame shift, creating a stable and potent transcription factor that induces a plethora of genes involved in protein folding, lipid synthesis (that increases ER volume) and translocation into the ER. Importantly, the ER‐associated degradation (ERAD) pathway that helps to remove misfolded proteins is also induced [29]. ATF6 is activated after cleavage by site 1 and site 2 proteases, and translocates to the nucleus to induce the expression of XBP1, BiP, CHOP and genes involved in ERAD [30]. For an in‐depth discussion of the roles of IRE1 and ATF6 in neurodegenerative disease, see Hetz and Mollereau [16].

## UPR activation in neurodegenerative disease

The UPR is central to the cell's response to dysregulated proteostasis. Markers of UPR activation including PERK‐P and eIF2α‐P have been reported in the brains of patients with AD, PD, ALS, the tauopathy progressive supranuclear palsy (PSP) and prion disease [31, 32, 33, 34, 35, 36]. Upregulation of UPR markers in the brain in these disorders are temporally and spatially associated with abnormal protein aggregation and the occurrence of neuropathological features. In ALS, the detection of ER stress markers in body fluids has even been suggested as a possible biomarker for disease progression [37]. Overactivation of the PERK branch of the UPR has recently been implicated in the pathogenesis of PSP, a tauopathy characterized by widespread tau pathology and progressive neurodegeneration. PERK‐P and eIF2α‐P are found in the pons, medulla, midbrain and hippocampus of *post mortem* patients, the regions of the brain most affected by PSP [34]. A genome‐wide association study searching for common variants influencing the risk of PSP found a single‐nucleotide polymorphism (SNP) in intron 2 of the PERK gene, *EIF2AK3* [38]. This SNP was found to be in linkage disequilibrium with three non‐synonymous coding variants, two in the ER luminal protein‐sensing domain and the third in the PERK kinase domain. The functional significance of these variants was previously investigated in lymphoblastoid cell lines [39]. When these cells were exposed to the ER stressor thapsigargin, they demonstrated a stronger stress response, suggesting the high‐risk variant increases the risk of PSP via a stronger UPR activation. Animal models of neurodegeneration also exhibit upregulation of ER stress markers, including rTg4510 mice, which overexpress the human tau mutation P301L associated with FTD, 5xFAD mice that express five AD linked mutations, mutant SOD1 (mSOD1) mice and a mutant huntingtin mouse model [40, 41, 42, 43].

## UPR in prion disease: cause and effect in neurodegeneration

There is an obvious correlation between UPR markers and neuropathology, but does UPR activation actively contribute to these diseases? Recently, we directly linked UPR activation to neurodegeneration in prion‐diseased mice. We used the Tg37^+/−^ mouse model of prion disease that succumbs to Rocky Mountain Laboratory prion infection in around 12 weeks [44, 45, 46]. Synapse loss occurs in these mice at 7 weeks post‐infection (wpi), followed by memory loss at 8 wpi, a drastic reduction in protein synthesis and synaptic protein levels at 9 wpi, and neuronal death at 10 wpi. Rising levels of misfolded prion protein cause sustained overactivation of the PERK‐eIF2α branch of the UPR in neurons resulting in an uncompensated decline in global translation rates, synaptic failure and neuronal death [47]. Similar UPR activation was also observed in wild‐type mice [47].

**Figure 2:**
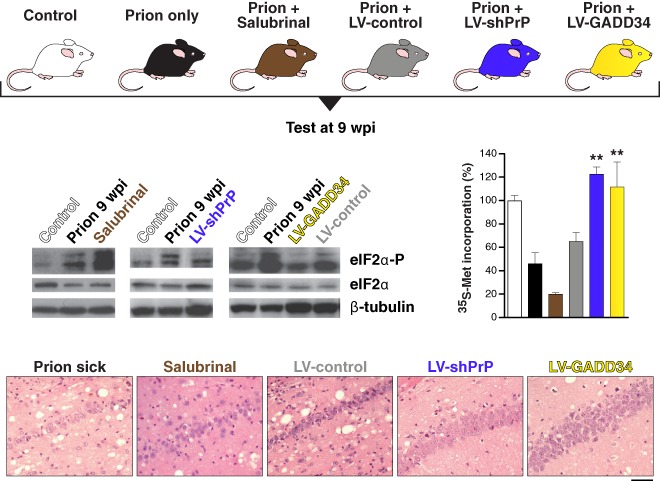
Manipulation of the UPR rescues protein translation and is neuroprotective in prion‐diseased mice. Lentivirally delivered shRNA against PrP (blue bar) or overexpression of GADD34 (yellow bar) reduces levels of eIF2α‐P, restoring global protein synthesis rates, as measured by ^35^S‐methionine incorporation levels, compared with uninfected mice (white), untreated prion‐diseased mice (black) or empty vector controls (grey). Salubrinal (brown) had a detrimental effect in the same experiments. The restoration of translation provided neuroprotection (panels). Bar chart shows mean ± standard error of the mean (**P < 0.01). Adapted from Moreno *et al*. [47]. LV, lentivirus; PrP, protease‐resistant prion protein; shRNA, short hairpin RNA; UPR, unfolded protein response; wpi, weeks post‐infection.

We first tested the hypothesis that reducing eIF2α‐P would restore translation and prevent neurodegeneration (Figure 2). Prion‐infected mice were injected with lentiviruses expressing either the eIF2α‐P‐specific phosphatase GADD34 or short hairpin RNA to reduce PrP expression. Salubrinal, an inhibitor of eIF2α‐P dephosphorylation, was also tested to determine if it exacerbated disease, as this increases eIF2α‐P [47].

Both GADD34 overexpression and PrP knockdown restored global translation rates at 9 wpi. As a result, synaptic protein levels, synaptic transmission and synapse number in prion‐diseased mice treated with GADD34 or PrP knockdown were protected and equivalent to levels in uninfected control mice. There was extensive neuronal protection in the hippocampus, with no neuronal loss and greatly reduced spongiform change. The burrowing behavioural phenotype, which measures motivation and is dependent on an intact hippocampus and pre‐frontal cortex [48, 49], was also protected. Overexpression of GADD34 and PrP knockdown also had a modest, but highly significant, effect on survival [47].

Critically, treatment with salubrinal had the opposite effect, by preventing dephosphorylation of eIF2α‐P. Thus, eIF2α‐P levels were markedly higher at 9 wpi than in prion‐only controls, causing further repression of global translation. Salubrinal treatment resulted in earlier severe neuronal loss and significantly accelerated disease compared with untreated prion‐infected mice [47].

**Figure 3:**
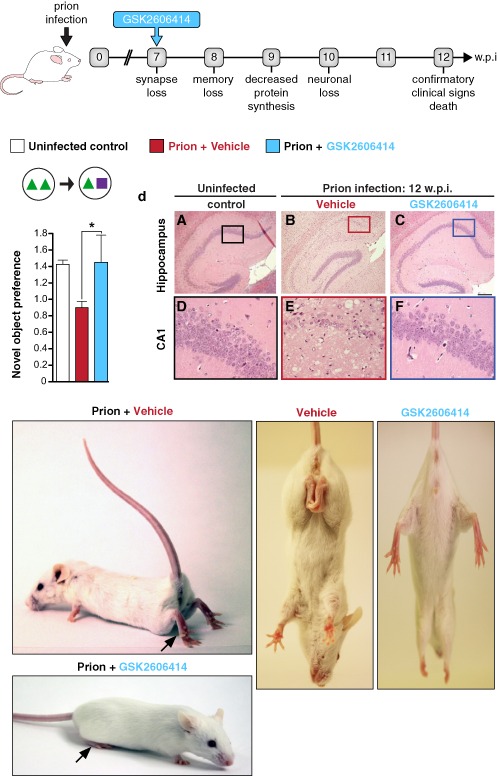
PERK inhibition by GSK2606414 prevents clinical disease in prion‐infected mice. Mice were treated with GSK2606414 (blue) or vehicle (red) from 7 wpi. GSK2606414 restored global protein synthesis rates and prevented loss of novel object memory, providing marked neuroprotection in the hippocampus. Pictures show clinical cure in treated mice with normal posture and movement of hind legs. Bar chart shows mean ± standard error of the mean. Controls represent uninfected mice (white bar) (*n* = 12 for each) (*P < 0.01). Adapted from [51]. PERK, PKR‐like endoplasmic reticulum kinase; wpi, weeks post‐infection.

The striking neuroprotection achieved by genetic manipulation of the UPR led to the prediction that pharmacological inhibition of PERK/eIF2α‐P would be similarly protective. A highly selective inhibitor of PERK, GSK2606414 [50], was tested in prion‐infected mice by oral administered from 7 wpi [51]. The PERK inhibitor prevented the rise of eIF2α‐P observed in disease and restored global protein synthesis rates. As with genetic manipulation of the UPR, there was marked neuroprotection throughout the brain and the absence of clinical signs of disease (Figure 3). The beneficial effects were also observed in animals treated after behavioural signs had emerged [51]. Effects on survival could not be assessed due to toxicity associated with the compound, which resulted in weight loss and mild hyperglycaemia, necessitating termination of the experiment. Critically, the compound acts downstream, and independently, of the primary pathogenic process of prion replication and is effective despite continuing accumulation of PrP. We hypothesize that the UPR is triggered by rising levels of total PrP synthesis in the ER rather than as a direct effect of aggregation of misfolded PrP as this occurs largely extracellularly or within the endosomal compartment. This stems from the observation that total PrP mRNA levels increase during prion infection, suggesting that increased synthesis of native PrP may cause misfolding and UPR activation [47], and there is evidence that overexpression of protein production can induce UPR markers [52].

There is further evidence for a role of the UPR in prion disease. Upregulation of several chaperones and ER stress proteins such as BiP, GRP94 and GRP58 is observed in patients with CJD, which is mirrored in mouse models of prion disease [35, 36]. This suggests ER stress and abnormal homeostasis are features of prion disease. Disruption of calcium homeostasis, and the resulting ER stress, has emerged as another component of the development of prion disease. Exposing cells to purified PrP^Sc^ from the brain of scrapie‐infected mice induces ER stress and the release of calcium from the ER. This is associated with the upregulation of several ER chaperones, which are also found in the brains of CJD patients [53]. Cells chronically infected with prions are more susceptible to ER stress‐mediated cell death, linked with a stronger UPR activation after exposure to ER stress‐inducing agents such as tunicamycin and thapsigargin [53].

GSK2606414 has also been used to ameliorate TDP‐43 toxicity in *Drosophila* and mammalian neuron models of ALS [54]. These beneficial effects were again due to the reduction of eIF2α‐P levels, and these data also demonstrate that PERK inhibition is likely to be useful against the range of neurodegenerative disorders. Genetic reduction of PERK has also been demonstrated to prevent neurodegeneration and cognitive impairment in the 5xFAD model of AD [43].

As well as the direct therapeutic interventions that target the UPR to prevent neurodegeneration discussed above, there is increasing evidence of the involvement of dysregulated UPR signalling across the spectrum of neurodegenerative disease.

## UPR in AD

In AD, eIF2α‐P levels correlate with elevated BACE1 (an enzyme that cleaves APP into Aβ) levels in transgenic mice as well as in AD patient brains [55]. A comparison of the expression of BiP in the different Braak stages of AD suggests UPR activation is an early event [56]. Treatment of cells with Aβ peptides leads to the activation of ER‐specific caspases, which correlates with the induction of apoptotic cell death [57]. Exposing cells to Aβ oligomers or fibrils in different experimental models can also trigger ER stress, which has been shown to lead to the phosphorylation of eIF2α, PERK and other indicators of UPR activation [58]. XBP1 controls a number of UPR‐related genes, but it has also been reported to upregulate a number of AD‐associated proteins, including CDK5, and the γ‐secretase complex, which is involved in APP processing to Aβ [59]. ATF4 has also been shown to regulate γ‐secretases, enhancing their activity during periods of UPR activation [60, 61]. ER stress may be inducing a positive feedback mechanism where protein aggregation may be exacerbating the conditions that promote the production of the misfolding protein itself. In agreement with this, PERK knockout significantly decreases Aβ load in the hippocampus [62]. This further emphasizes the potential of UPR inhibition as a promising therapeutic target.

UPR activation is also associated with hyperphosphorylated tau. PERK‐P has been observed in neurons and glia that exhibit tau pathology, and is upregulated during the early phase of disease [63]. In vitro studies suggest that induction of ER stress by the exposure of cells to Aβ oligomers correlates with the induction of tau phosphorylation, suggesting a link between ER stress, Aβ‐mediated neurotoxicity and tau hyperphosphorylation [64]. Induction of UPR signalling has been shown to induce tau phosphorylation, possibly via the activation of glycogen synthase kinase 3β (GSK‐3β) [65], demonstrating a direct link between UPR activation and neurodegenerative processes. Furthermore, neurons displaying PERK‐P coexpress active GSK‐3β in AD brains [32]. The link between metabolic stress and UPR activation caused by tau phosphorylation has been investigated [66]. It was found that metabolic stress activated the UPR, which in turn led to the reversible phosphorylation of tau. Inhibiting the UPR using GSK2606414 and reducing cellular stress with a chemical chaperone reduced tau phosphorylation, validating UPR inhibition in tauopathies [66]. Studies by Ma *et al*. and Lourenco *et al*., discussed in more detail below, also provide evidence for a direct role of the UPR in AD [62, 67].

## UPR in PD

In PD, mutant α‐synuclein has also been shown to accumulate within the ER, directly activating the PERK arm of the UPR by binding to BiP [68]. The accumulation of mutant α‐synuclein in dopaminergic cells increased the expression of BiP and induced the expression of the UPR‐related transcription factor ATF4. The authors also suggested that activation of the UPR pathway in cells by mutant α‐synuclein coincided with pro‐apoptotic changes [68]. The A53T missense mutation in the α‐synuclein gene causes dominant familial PD [69]. This mutation is associated with UPR activation, as observed by an increase in CHOP and BiP expression and increased phosphorylation of eIF2α, suggesting the UPR is active in these cells [70]. ER stress leads to mitochondrial dysfunction, but inhibition of caspase‐12, a caspase induced by UPR activation, protected the A53T α‐synuclein‐overexpressing cells from cell death, suggesting that the activated UPR was inducing apoptosis [70].

## UPR in ALS

Approximately 2% of ALS patients have a mutation in the SOD1 gene and transgenic rodents expressing the mSOD1 are the most commonly used model of study in ALS research [71]. mSOD1 misfolds, aggregates and induces the UPR in transgenic mSOD1 mice, causing apoptosis and has been implicated in the development of ALS [72]. The levels of the ER chaperone, protein disulphide isomerase (PDI) in particular, were increased and were shown to co‐localize with aggregated mSOD1 protein. In a study using mSOD1 mouse models of ALS, vulnerable motor neurons were shown to be selectively prone to axonal degeneration in cells that demonstrated a UPR response. This could be attenuated or exacerbated by treatment protecting against or stimulating further ER stress, respectively [40]. Insufficient ERAD of misfolded proteins is associated with a range of neurodegenerative conditions, including ALS. The dysfunction of ERAD, causing ER stress, has been shown to occur in mSOD1‐containing motor neurons, through a mechanism involving Derilin‐1, an ERAD‐linked protein, subsequent ER stress‐induced activation of the ASK1 pathway and ultimately apoptosis [73]. This was found to be caused by the interaction between Derilin‐1 and mSOD1, which caused dysregulation of ERAD leading to ER stress‐induced ASK1 activation, apoptosis and disease progression.

## ER stress or other processes? Conflicting evidence involving the UPR

Although the UPR is normally a protective cellular response, the data presented above demonstrate that its dysregulation has a central role in many diseases. Chronic UPR activation, and the resulting swith from cytoprotective to cytotoxic signalling, contributes to disease. There are a number of unresolved questions, including: Why does the UPR become chronically activated and what causes the switch to cytotoxic signalling? If the majority of the disease‐modifying proteins discussed above do not build up in the ER lumen, why is the UPR activated? Is ER stress a response to neurodegeneration or does it contribute to disease initiation?

These questions are now beginning to be answered. In most cases, it is likely that the induction of ER stress in neurodegenerative disease is not caused by misfolded protein build up in the ER lumen. However, some examples exist where disease‐associated misfolded proteins do build up in the ER. mSOD1 can translocate into the ER and has been observed bound to BiP and the foldase PDI [72]. Mutant α‐synuclein oligomers have also been found inside the ER in animal models as well as human tissue samples, again in association with BiP [68, 74]. These misfolded proteins may be sequestering essential ER proteins such as BiP, triggering ER stress. Interestingly, in mSOD1 and mutant α‐synuclein models of neurodegeneration, treatment with salubrinal (hence increasing eIF2α‐P levels) is protective, in contrast to prion disease where salubrinal exacerbates disease 4 [40, 75]. When mSOD1 and mutant α‐synuclein aggregate in the ER, they are triggering a form of ‘pure’ ER stress that the UPR is designed to combat, so it is no surprise that augmenting UPR activity with salubrinal is neuroprotective. However, in other diseases such as AD, the tauopathies and prion disease, where misfolded protein aggregation is mainly cytoplasmic or extracellular, there is a more general nonspecific proteostatic dysregulation, hence relief of ER stress itself is less relevant than restoring protein synthesis. It is here that chronic UPR activation becomes detrimental to the cell and UPR inhibition is a viable therapeutic target. This leads to the hypothesis that the secondary effects of translational shutdown is the lethal process driving neurodegeneration in these cases, hence why reducing eIF2α‐P levels is neuroprotective.

As discussed above, it is possible that the increased transcription of PrP, rather than the buildup of misfolded PrP^Sc^ itself, is a cause of the ER stress in prion disease. Further investigations are also uncovering mechanisms of UPR activation caused by other misfolded proteins that do not build up in the ER. Abisambra *et al*. investigated how tau can activate the UPR using the tg4510 mouse model and an inducible tau cell model [41]. They found increasing PERK‐P as the disease progressed, which was preceded by an increase in ubiquitin. ERAD tags unfolded proteins with ubiquitin, which targets them for degradation by the proteasome. Removing tau caused the levels of PERK‐P and ubiquitin to lower. Tau was found to co‐immunoprecipitate with components of the ERAD system that export misfolded proteins from the ER, and proteins that are normally efficiently removed by ERAD built up in the hippocampus of tau positive mice. The authors conclude that tau was able to impair ERAD directly, leading to UPR stress and the associated cell death that occurs when this pathway is activated. Mutant huntingtin, the misfolded protein associated with Huntington's disease, has also been shown to cause stress via the blocking of the ERAD pathway [76]. However, the precise molecular steps between ERAD disruption and subsequent eIF2α phosphorylation are unclear.

## Memory: another key role for protein synthesis

Protein synthesis is also central to memory formation, particularly via eIF2α modulation. This is important in this context, as memory loss is very often a central feature of neurodegenerative disease, especially in dementia. Neurophysiologically, memories are usually divided into short‐term memory, typically classed as lasting only a few hours in duration, and long‐term memory, lasting for years or even the lifespan of the organism, but there is no absolute distinction between memory types [77]. Long‐term memories require *de novo* protein synthesis [78], but there are no definitive time frames that differentiate protein synthesis‐dependent and protein synthesis‐independent memories. Memory is often described as an activity‐dependent change in the strength and/or number of synaptic connections that underpin long‐term changes in neural circuits with a possible adaption in behaviour [79]. The hippocampus and the amygdala are the two most important structures in the brain involved in memory. Widely studied correlates of memory are long‐term potentiation (LTP) and long‐term depression as they share similar molecular and cellular mechanisms [80]. Longer lived forms of LTP also require protein synthesis [81]. Conversely, protein synthesis inhibitors have repeatedly been shown to inhibit memory formation and consolidation in multiple behavioural models [82]. The use or retrieval of an established memory also results in a second phase of increased protein synthesis, a process referred to as memory reconsolidation [83].

**Figure 4:**
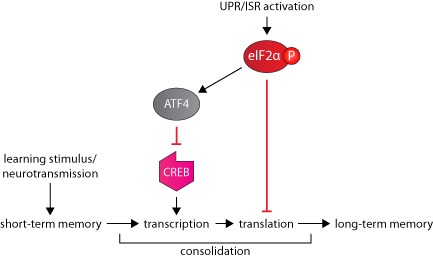
The role of the UPR in long‐term memory formation. A learning stimulus leads to short‐term memory formation. The process of consolidation, which requires transcription and translation, cements short‐term memories into long‐term memories. The UPR can inhibit this process via eIF2α‐P‐mediated translational repression and inhibition of the transcription factor CREB by ATF4. ATF4, activating transcription factor 4; CREB, cAMP response element‐binding protein; eIF2α‐P, phosphorylated eIF2α; ISR, integrated stress response; UPR, unfolded protein response.

So how is the UPR involved in memory formation and consolidation? The translational attenuation of protein synthesis after eIF2α phosphorylation will prevent many of the proteins required from being produced during periods of ER stress that are required for LTP and long‐term memory formation. Although dendrites have a pool of mRNA and translational machinery at the synapse, this is not resistant to eIF2α‐P, and any longer term memory consolidation or reconsolidation will require *de novo* protein synthesis. Significantly, ATF4 is a repressor of cAMP response element‐binding protein (CREB)‐mediated gene expression, which is essential for long‐lasting changes in synaptic plasticity and memory [84]. Consequently, eIF2α phosphorylation impinges on two central processes that are crucial for the formation and storage of long‐term memories: new protein synthesis and CREB‐mediated gene expression (Figure 4). In agreement with this, stimuli that produce increases in synaptic strength, such as tetanic stimulation, brain‐derived neurotrophic factor or cAMP activators, decrease the phosphorylation of eIF2α [85, 86]. In a fear conditioning test that directly induces protein synthesis‐dependent memory, eIF2α‐P is also reduced, leading the authors to conclude that eIF2α regulates the switch from short‐ to long‐term synaptic plasticity and memory [87].

## Modulating the UPR to enhance memory

Protein synthesis is therefore an essential part of memory storage, and the UPR tightly regulates protein synthesis rates, especially during periods of stress. This presents the question: can modulating the UPR enhance memory? Genetic reduction of eIF2α‐P in hippocampal slices from mice, either lacking GCN2 or heterozygous for a mutated eIF2α that cannot be phosphorylated, reduced the threshold for the induction of both LTP and learning in several behavioral tests [85, 87]. Conversely, preventing eIF2α dephosphorylation with the small molecule Sal003 blocks both LTP and long‐term memory formation. The impairment of LTP by Sal003 is mediated by ATF4, as LTP induced in hippocampal slices from ATF4 knockout mice is resistant to Sal003 [87]. Exacerbated phosphorylation of eIF2α was also observed to induce cognitive impairment [88]. In agreement with these findings, a study demonstrated that genetic deletion of two of the eIF2α kinases, PERK and GCN2, improves cognitive function and synaptic plasticity [62]. PKR, another eIF2α kinase, has also been deleted, which was also shown to increase learning and memory [89].

Several recent studies have explored the link between UPR activation, memory and neurodegeneration. Lourenco *et al*. demonstrated that brain inflammation in AD models engages PKR to induce synaptic loss and memory impairments [67]. The authors also demonstrated that Aβ oligomers alter insulin signalling leading to memory deficits through a mechanism involving the pro‐inflammatory cytokine tumor necrosis factor‐α. After investigating the effects of PERK and GCN2 knockout, Ma *et al*. also demonstrated that these deletions can protect against memory impairment and neurodegeneration in AD model mice [62]. Genetic deletion of PERK and administration of a small molecule PERK inhibitor improved memory deficits as well as offering substantial neuroprotection [43, 51]. Importantly, these reports demonstrate that despite reducing the activity of one branch of the UPR, genetic or pharmacological manipulation improved cognitive aspects of neurodegenerative disease without affecting the ability of the neurons to survive the stress of misfolded protein aggregation.

It is unclear if the improvements in memory after UPR inhibition in these disease models are due to the release of translational repression that was preventing memory formation, or a consequence of the neuroprotection conferred. However, it is possible to improve memory using small molecules targeting downstream UPR signalling. An interesting study identified a small molecule, ISRIB, that inhibits downstream signalling of eIF2α‐P [90]. ISRIB improves memory in mice and rats in a number of behavioural tests, without directly lowering eIF2α‐P levels. However, it effectively makes cells insensitive to eIF2α–P, lowering ATF4 expression. The authors conclude that memory consolidation is inherently inhibited by eIF2α phosphorylation, and compounds such as ISRIB can release cells from this tonic inhibition.

## Conclusions

The UPR is the focus of increasing interest as an attractive target for disease‐modifying novel therapies due to its emerging role in the pathogenesis of neurodegenerative disease. The fact that these therapies are likely to increase memory in patients, where memory loss is a major problem, further improves the attractiveness of the UPR as a target. It remains to be seen if the problems associated with PERK inhibition can be abrogated. This, and other agents that mimic the action of ISRIB, for example, might hold the key to the first truly disease‐modifying and nonspecific treatment for neurodegeneration. Further work is needed to elucidate the exact nature of how these misfolded proteins cause ER stress and UPR activation, and the conditions that push UPR activation from a beneficial protective response to a central tenet of disease pathogenesis. UPR activation may also provide a useful biomarker for neurodegenerative disease.

In conclusion, it is becoming increasingly clear that the translational repression due to phosphorylation of eIF2α is a key link between neuropathology and memory impairment in neurodegenerative disease. The reports discussed in this review suggest that restoration of protein synthesis through the eIF2α pathway is an attractive target to reduce memory impairment and prevent neurodegeneration across the spectrum of these disorders.
